# Creation of Numerical Constants in Robust Gene Expression Programming

**DOI:** 10.3390/e20100756

**Published:** 2018-10-01

**Authors:** Iztok Fajfar, Tadej Tuma

**Affiliations:** University of Ljubljana, Faculty of Electrical Engineering, Tržaška 25, 1000 Ljubljana, Slovenia

**Keywords:** genetic programming, gene expression programming, genetic algorithms, genotype/phenotype evolutionary algorithms, symbolic regression, constant creation, ephemeral random constants, numeric mutation, numeric crossover, digit-wise crossover

## Abstract

The problem of the creation of numerical constants has haunted the Genetic Programming (GP) community for a long time and is still considered one of the principal open research issues. Many problems tackled by GP include finding mathematical formulas, which often contain numerical constants. It is, however, a great challenge for GP to create highly accurate constants as their values are normally continuous, while GP is intrinsically suited for combinatorial optimization. The prevailing attempts to resolve this issue either employ separate real-valued local optimizers or special numeric mutations. While the former yield better accuracy than the latter, they add to implementation complexity and significantly increase computational cost. In this paper, we propose a special numeric crossover operator for use with Robust Gene Expression Programming (RGEP). RGEP is a type of genotype/phenotype evolutionary algorithm closely related to GP, but employing linear chromosomes. Using normalized least squares error as a fitness measure, we show that the proposed operator is significantly better in finding highly accurate solutions than the existing numeric mutation operators on several symbolic regression problems. Another two important advantages of the proposed operator are that it is extremely simple to implement, and it comes at no additional computational cost. The latter is true because the operator is integrated into an existing crossover operator and does not call for an additional cost function evaluation.

## 1. Introduction

Gene Expression Programming (GEP) [1] is a relatively new, but established type of genotype/phenotype evolutionary algorithm closely related to Genetic Algorithms (GA) and Genetic Programming (GP). The elementary difference between GA, GP and GEP lies in the nature of the encoding of individuals: the individuals in GAs are encoded as linear strings of fixed length; the individuals in GPs are nonlinear entities of various sizes and shapes (most usually trees); and the individuals in GEPs are linear strings of fixed length, which are then expressed as trees. GEP can be used to solve different problems through the evolution of computer programs (phenotype), which are encoded in the form of linear gene expression strings (or chromosomes) of fixed length (genotype). During the evolution process, the chromosomes are manipulated by means of nature-inspired operations like mutation and crossover. Since its inception, GEP has attracted an increasing number of researchers that have proposed innumerable enhancements to the basic concept. Presently, GEP is considered an effective tool for searching for programs to solve real-world problems from many fields of science and engineering. These include, but are not limited to, data mining, time series prediction, classification and regression problems and knowledge discovery (see, for example, ref. [2] and the references therein).

Many problems that are usually attacked by GEP include finding mathematical formulas, the integral part of which are numerical constants. In order to be able to produce highly accurate solutions, it is therefore essential for GEP to be able to create numerical constants. However, it is a great challenge for GEP to create highly accurate constant values as these values are continuous, while the GEP chromosome representation is suited for combinatorial optimization. Very recently, the constant creation problem has been defined as one of the critical open research issues [2].

Not only in GEP, but in the GP community in general, the most commonly utilized constant creation method is the Ephemeral Random Constant (ERC) technique proposed by Koza [3]. This method simply includes constants as ordinary terminals, whose values are initially chosen at random within a specific range of values. After that, these constants move between (and within) expression trees through crossover operations, or combine with available arithmetic operators, thus producing new constants. This simple approach, however, has difficulties discovering even a single numeric constant [3]. Soon after publishing the original GEP concept [1], Ferreira proposed a special constant creation method for GEP [4]. The approach initially generates a separate vector of constants and appends a special Dcdomain containing indices of constants in the constant vector after the tail of the chromosome. However, Ferreira also discovered that GEP performed considerably better without using the proposed constant creation technique [4], simply because using numerical constants disproportionately increases the search space. Peng et al. improved Ferreira’s approach by including some pre-selected user-defined constants, as well as small random floating point constants used for fine-tuning the solutions [5]. While their constant creation approach was better than that of Ferreira, it showed only a slight improvement over the original GEP. Many authors experimented with different numeric mutations (e.g., [6,7,8,9]), which are slight variations of two basic mutation types, namely creep mutation and random mutation. Creep mutation selects a random constant gene and changes its value by a small random value bounded by a pre-defined creep step. Random mutation, on the other hand, replaces the value of a gene with a completely new random value, chosen between the prescribed bounds for that gene. It has not been clearly decided which of the two mutation types is better as their behavior depends on the problem at hand, but both are significantly better than the basic ERC technique. Another typical approach to the constant creation problem uses a separate real-valued optimization method to fine-tune constants of individuals in each generation. Any method capable of solving continuous optimization problems is suitable for this task, although authors prefer to employ Evolutionary Algorithms (EA), such as differential evolution [10,11,12] or particle swarm optimization [13]. All in all, EA-based algorithms are more capable of finding highly accurate constants than their mutation-based counterparts, but, at the same time, are more difficult to implement and are computationally quite expensive. As a consequence, many authors still prefer to use the original approach of Koza (e.g., [14,15]), as it yields the best results at the least cost.

One quite different approach can be found in [16], where the authors propose a special concatenation grammar to be used in Grammatical Evolution (GE) systems. The method proved to be very successful in constantly introducing new constants to a system. Nevertheless, due to implementation specificity, it has only been used in GE.

In this paper, we employ Robust GEP (RGEP) [17], which is a simplification of GEP. Like GEP, RGEP also encodes trees, but requires neither building actual trees, nor encoding any knowledge about the eventual derivation trees in the genetic operators. Our main contribution is a simple numeric crossover operator to be used instead of numeric mutation, which, at no additional computational expense, and with quite a minimal implementation effort, significantly improves the constant creation ability of the original RGEP. We successfully tested it on 21 specially-devised symbolic regression problems containing one or two five-digit constants and on two standard benchmark functions. Partly, the proposed numeric crossover operator resembles the mechanism from [16] in that it manipulates constants on a digit level, but the implementation and basic operation principle are quite different because of different encoding strategies of GE and RGEP. Secondly, we show that one can further improve the efficacy of the RGEP algorithm by constraining types of certain genes within the chromosome, thus increasing the locality in genotype/phenotype transformation.

The paper is organized as follows. In the next section, we summarize the original RGEP algorithm with some important implementation guidelines, while the proposed improvements to the original algorithm are detailed in Section 3. The setup of the experiment, together with the selected genetic parameters and symbolic regression formulas, is outlined in Section 4. Finally, the results are presented and discussed in Section 5 and Section 6, respectively.

## 2. Robust GEP

For the sake of convenience and clarity, we first briefly outline the main idea behind the concept of RGEP [17] and add important implementation details. The main advantage of RGEP over the standard GEP is that it uses prefix notation for its genotype representation [18]. This results in less disruptive genetic operations as there is greater locality in the genotype/phenotype transformation. The second advantage of RGEP is its special evaluation method, which allows much simpler chromosome encoding, without any need for separate head and tail sections. This simplification also significantly simplifies basic genetic operators, for they do not have to respect the differences in the structure of the head and tail of the chromosome as they have to in the original GEP.

### 2.1. Chromosome Evaluation

Conceptually, there is no difference between prefix and postfix notation, but as soon as one uses a stack to evaluate expressions, postfix notation comes more natural. For this reason, we use postfix notation (also known as Reverse Polish Notation (RPN)) to evaluate our chromosomes, and the whole evaluation process is outlined in Algorithm 1. Notice that the evaluation process follows the standard postfix evaluation rules with additional error checking: operators in the chromosome are executed if and only if there are enough operands on the stack. Otherwise, if there were not enough operands available, the operator is simply ignored, and the evaluation continues with the next gene from the chromosome. Notice that this kind of encoding and evaluation ensures that no invalid individual can appear during the evolution, except in an extremely unlikely case that all genes within the chromosome are operators. In that case, the evaluation simply returns the worst possible fitness, and such an individual will soon disappear from the population. Another advantage of the described encoding is that no tree has to be built, which simplifies the implementation immensely.

**Algorithm 1:** RGEP chromosome evaluation procedure.**for** Each gene in chromosome **do** **if** The gene is an operator **then**  **if** Number of elements on stack ≥ arity of the operator **then**   Pop necessary operands from stack   Execute the operator   Push the result back on stack  **end if** **else**  //Comment: the gene is an operand  Push the operand on stack **end if**
**end for**
The topmost element on the stack is the value of the evaluated expression.

As an example, consider the following chromosome:

(1) {3,8,+,∗,x,sin,x}.

According to Algorithm 1, numbers 3 and 8are first pushed onto the stack. Then, in order to execute the addition operator, 8 and 3 are popped from the stack, added together, and the result is pushed back onto the stack. The multiplication operator, which comes next, is ignored, for there is now only a single value on the stack (i.e., 11). After that, the variable *x* is pushed onto the stack, and the remaining two (unary) operators are both applied to this variable. Finally, we end up with two values on the stack: 11 and $\sqrt{\sin(x)}$, the second of which is the result of our evaluation.

### 2.2. Genetic Operators

Following the simple linear encoding of a chromosome, the implemented genetic operators are very simple, as well. The initialization of an individual is carried out by randomly selecting genes from a user-defined pool of primitives, containing constants and variables (also known as terminals) and operators (also known as functions). In the original paper, there was no clear guidance for how to select the primitives. However, in order to achieve the best possible function to terminal ratio, we compute the probability of selecting a terminal as:

(2) $$p_{T}=p_{F}\left(1+\sum_{i=1}^{n}\left(A_{i}-1\right)\right)/n,$$

where $p_{F}$ is a probability of selecting a function and *n* is the number of functions in the primitive set. $A_{i}$ is the arity of the *i*th function. The formula was derived from the fact that each new function inserted in the derivation tree calls for A−1 new terminals. Of course, the sum of both probabilities must be $p_{T}+p_{F}=1$. In the case of selecting a terminal, a further selection should be made, namely between a variable and a constant. We have:

(3) $$p_{T}=p_{\mathrm{Tc}}+d\cdot p_{\mathrm{Tv}},$$

where $p_{\mathrm{Tc}}$ is the probability of selecting a constant and $p_{\mathrm{Tv}}$ the probability of selecting any of *d* available variables. Note that, whenever a constant is selected, its value is generated at random within user-defined limits and can be modified later only through mutation.

According to the analogy with GAs, our mutation is akin to uniform mutation of a single randomly-selected gene. The only difference from the uniform mutation is that we replace the selected gene with a gene randomly chosen from the pool of primitives according to the probabilities (2) and (3). In a similar vein, we use one- or two-point crossover from GAs, where crossover points are selected at gene instead of bit positions.

The only operator that is not commonly used in GAs is rotation. This operator is implemented as a right shift of a chromosome for an arbitrary number of genes with wrapping at the end. Rotation is an important operation as it enables the exchange of subtrees between different parts of the parse tree.

**Figure 1:**
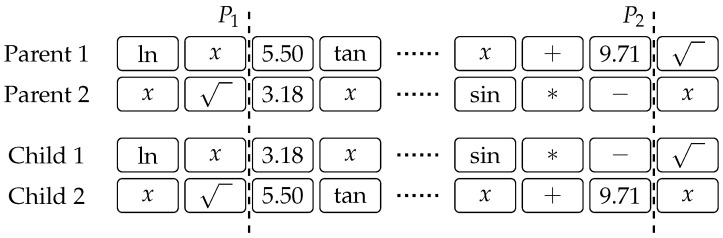
Two-point crossover.

**Figure 2:**
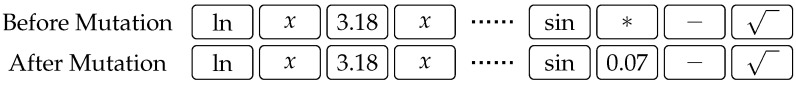
Mutation.

**Figure 3:**
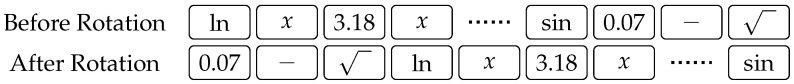
Rotation.

Figure 1 shows an example of two-point crossover, where the genetic material is exchanged between two parents. The portions of chromosomes between points $P_{1}$ and $P_{2}$ are swapped in order to produce two offspring. An example of mutation is shown in Figure 2, where the third gene from the right is randomly mutated. There is no limit on which of the available primitives is picked as a replacement of the gene that has been selected for mutation, and a new gene is selected according to the probabilities given by (2) and (3). Finally, Figure 3 illustrates an example of rotating a chromosome by three places to the right. Notice how genes at the end of the chromosome wrap to the beginning.

## 3. The Improvements

In this section, we propose two modifications to the original RGEP algorithm and give their implementation details.

### 3.1. Numeric Crossover

**Figure 4:**
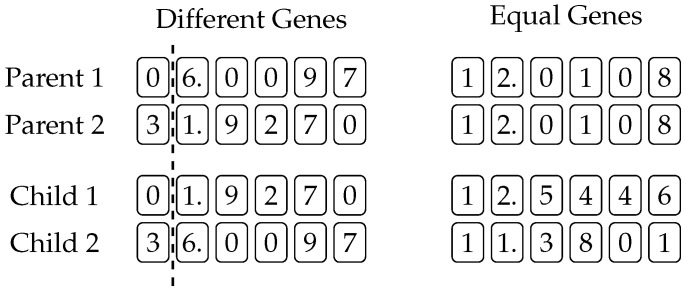
Proposed numeric crossover.

The first modification is the addition of a numeric crossover operator, which augments the original crossover mechanism from Figure 1. This operator is executed every time two constant genes get aligned, as, for example, constant values 5.50 and 3.18 in Figure 1. In the original (one- or two-point) crossover, the aligned constant genes are simply swapped, just like genes of any other type. Our crossover operator, however, carries out an additional digit-wise crossover of the aligned constants, as shown in Figure 4. If the aligned genes are different, then a normal one-point crossover is performed as shown at the left-hand side of Figure 4: A crossover point is randomly selected, and the digits following that point are swapped. If, however, the genes are equal, then the corresponding genes of the children are obtained by changing the value of the parents’ gene by a random value no greater than 10% of the gene’s original value. Note that this change is possible in either direction, and the example at the right of Figure 4 shows how the value of the constant gene of the first child is a little larger, while that of the second child is a little smaller than the original.

This second part of the proposed numeric crossover mechanism is akin to the local hill-climbing search operator proposed by [19], where a constant value is increased/decreased in successive 10% steps as long as it results in fitness improvement. Because this procedure represents a significant computational strain on the process, it is probabilistically applied in [19]. Our version of this hill-climbing operator, however, does not require any additional computation, as it is a part of the regular crossover operation. More precisely, the numeric crossover operator only burdens the execution time of the regular crossover operation, which is negligible considering the costly fitness evaluation that follows each crossover, and rises exponentially with the depth of the derivation tree.

### 3.2. Gene Constraint

Although the original coding scheme of RGEP as proposed in [17] results in less disruptive genetic operators than the original GEP, we discovered that RGEP still seriously suffers from the problem of locality. This is a well-known issue with genotype/phenotype evolutionary systems where many neighboring genotypes do not correspond to neighboring phenotypes [20,21]. One can easily imagine a simple gene mutation where, for example, a gene experiences a conversion from operand to operator. If this conversion takes place at the beginning of the chromosome, then the operators that follow will act on completely different operands. For this reason, we propose a special chromosome building scheme where each gene position is constrained either to hold a terminal or a function, but not both. The approach was motivated by the model proposed by Korns [22]. Korns used an assemblage of strings (chromosomes) that encode functions, constants and variables separately from the main (abstract) s-expression and linked those chromosomes by gene positions and a special term chromosome. We adapted this complex encoding in a way that it can be used with a standard RGEP algorithm. Algorithm 2 shows the building scheme for a chromosome representing a full derivation tree of depth *N*. The algorithm builds a chromosome template composed of T’s and F’s denoting the positions of terminals and functions, respectively. It starts out with a root node (represented by a single T) and, then, for each new tree level, duplicates the complete template and adds an extra function node at the end. Note that a plus sign (+) in the algorithm denotes string concatenation. Algorithm 2 assumes that all the operators have an arity of two, but it can readily be extended to accommodate operators of higher arity, as well. In fact, the number of replications of *template* on the right side of the assignment operator inside the for loop of Algorithm 2 should be equal to the maximum arity of any operator used. Furthermore, an operator with smaller arity can easily be inserted into a chromosome, in which case only the necessary number of operands is used as its arguments, while the surplus operands are simply ignored.

**Algorithm 2:** Generation of a chromosome template.**Input:***N*, the depth of the derivation tree*template* = T**for**i=1 to *N*
**do** *template* = *template* + *template* + F
**end for**


The following is an example of a chromosome respecting the gene constraint (using N=3), containing binary, as well as unary operators:

(4) {x,x,sin,9.1,x,sin,∗,x,0.1,+,x,1.7,pass,pass,+},

where pass is a special binary operator, which simply passes the first of its arguments unaltered to its superordinate node and discards the second one. The use of this operator enables us to encode arbitrary s-expressions with fixed-length chromosomes, including the smallest ones.

The chromosome is evaluated in a standard postfix fashion, except that two operands are always popped from the stack, regardless of the arity of the operator. If the arity of the operator is one, then the second of the popped operands is discarded. Algorithm 3 summarizes the evaluation procedure.

Following Algorithm 3, chromosome (4) evaluates to:

(5) $$\sin^{2}\left(x\right)+1.7.$$

It is not hard to see that the thus constrained chromosome encoding possesses a high degree of locality. If, for example, one changes any of the first three genes in the chromosome, this does not affect the evaluation of the rest of the chromosome.

Notice that, in the case of gene constraint, the crossover operator as described so far can still be used without any alternation, while the mutation operator calls for a minor modification. In order to maintain the required chromosome structure, only a terminal gene is allowed to replace a terminal gene, and only a function gene is allowed to replace a function gene. This limitation should be observed when randomly selecting genes for mutation.

**Algorithm 3:** Modified chromosome evaluation procedure.**for** Each gene in chromosome **do** **if** The gene is an operator **then**  Pop 2 operands from stack  **if** Arity of the operator <2
**then**   Discard the 2^nd^ operand  **end if**  Execute the operation  Push the result back on stack **else**  //Comment: the gene is an operand  Push the operand on stack **end if**
**end for**
The element left on the stack is the value of the evaluated expression.

**Figure 5:**
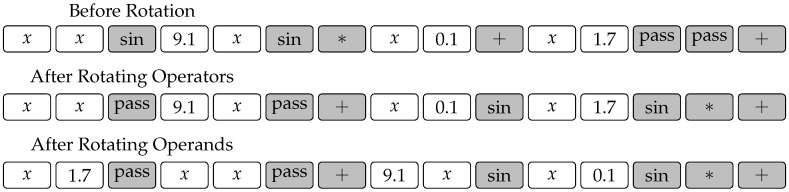
Modified rotation operator for use with a gene constraint where operator and operand genes are rotated separately. The operator genes are shaded for clarity.

The situation is quite different, however, with the rotation operator. The operator shown in Figure 3 would destroy the constrained chromosome in a way where the gene positions would not adhere to the template any more. Hence, we modify this operator as shown in Figure 5. The modified operator is in fact composed of two random rotations, the first of which rotates operator genes, while the second one rotates operand genes. Note that both rotations need not be carried out by the same number of spaces. Figure 5 shows an example where the operators and operands in chromosome (4) are rotated by three and two spaces, respectively.

Evaluation of the rotated chromosome from Figure 5 yields the expression:

(6) sin(0.1)sin(x)+x+1.7.

## 4. Experimental Setup

Table 1 shows the parameters that we used for our evolution runs. We determined these parameters based on related reports found in the literature, our previous experience and some preliminary experiments. It is important to note that we tuned the parameters trying to get the best possible performance, not using our numerical crossover lest we bias the parameters towards getting better improvements over existing methods. For the reason of more transparent comparison, we did not use any other stopping criteria than a fixed maximum number of generations. During the preliminary experiments, we discovered that less than a few percent of changes relevant to our research happen after 200 generations, and so, we settled with this number as a stopping criterion.

The primitive set (see Table 1) from which the process draws functions (Function Set) and terminals (Terminal Set) is different for single- and bi-variable problems in that the Terminal Set for bivariable problems contains an extra terminal (i.e., *y*). In the Function Set, there can be found some protected operators. It is a common practice in GP to use a protected version of an operator whenever there is the danger of an invalid operation like, for example, division by zero. We use the following three protected operators in our experiments:

(7) $$\begin{array}{ccc} x/y & := & \left\{\begin{array}{cc} x/y & \;\mathrm{if}\;y\neq 0,\\ 1 & \;\mathrm{otherwise}. \end{array}\right.\;\left(\mathrm{Protected}\;\mathrm{Division}\right) \end{array}$$

(8) $$\begin{array}{ccc} \ln(x) & := & \left\{\begin{array}{cc} \ln(x) & \;\;\;\mathrm{if}\;x>0,\\ 0 & \;\;\;\mathrm{otherwise}. \end{array}\right.\;\left(\mathrm{Protected}\;\mathrm{Natural}\;\mathrm{Logarithm}\right) \end{array}$$

(9) $$\begin{array}{ccc} \sqrt{x} & := & \sqrt{\left|x\right|}\;\;\;\;\;\;\;\;\;\left(\mathrm{Protected}\;\mathrm{Square}\;\mathrm{Root}\right) \end{array}$$

A value of the constant random terminal ($C_{\mathrm{random}}$) included in the Terminal Set is created randomly each time a constant terminal is selected, but is limited to the interval [0,10]. We set the probability of selecting a constant to $0.3\;p_{T}$, which means that approximately 30% of terminals will be constants.

It is important to distinguish numeric mutation (random or creep) from ordinary mutation. Ordinary mutation replaces a randomly-selected gene with any (randomly selected) primitive from the primitive set, observing the probabilities (2) and (3), as well as gene constraint (where applicable). Numeric mutation, however, is performed on all constant genes of the selected chromosome. Random numeric mutation replaces a numeric constant with a random constant uniformly selected between zero and 10, while creep numeric mutation changes a constant value by a uniform random value no greater than 10% of its original value. We applied numeric mutation at the end of each generation with the uniform probability of 0.2.

The algorithm was steady-state, and while we picked individuals for any type of mutation uniformly from the population, we selected the parents for crossover with tournament selection of size three and candidates for replacements with tournament selection of size two.

Finally, we used the Normalized Least Squares Error (NLSE) as a fitness measure (lower is better).

Because our main goal was to test the constant creation ability of different approaches, we decided to use the well-known problem of finding a mathematical formula that would model the provided data as accurately as possible. This task is known under the term Symbolic Regression (SR) and has troubled GP and its derivatives for a long time, mainly for the difficulty that GP has with producing accurate real-valued constants, as already mentioned. For the purpose of testing the proposed numeric crossover, we devised a special set of 21 formulas (i.e., SR problems) containing one or two five-digit real constants on which we tested our constant creation technique. We limited our problems to the maximum depth of three grammar nodes, so that all are solvable using chromosomes of length 15. Table 2 shows the collection of the test problems, many of which are very hard to solve for the plain RGEP, even when ephemeral constants are used in combination with either one of the two numeric mutations. During the evolution, we used 100 random points for calculating the fitness score (i.e., NLSE) from the corresponding interval indicated in the table. To eliminate possible cases that overfitted the training data, we used 100 different random points from the same interval to test the solutions in the next section.

## 5. Results

In this section, we report the results of running standard RGEP, as well as RGEP with a gene constraint (i.e., Constrained RGEP (C-RGEP)) on the 21 problems listed in Table 2. In particular, we are interested in comparing the variants with the proposed numeric crossover to the variants using either one of the two numeric mutations. Table 3 shows the results for the standard RGEP, reporting the obtained test NLSEs and *p*-values of statistical tests. In the three columns of the table following the column with the formula IDs are the results obtained by using three different numeric operators. For every target formula, there is a row showing the median test NLSE values, each one obtained by 50 independent evolution runs using the corresponding numeric operator. In the second row of each target formula (i.e., in parentheses below the median NLSE values), there are pairs of integers reporting the numbers of times when the best solution’s NLSE was better than $10^{-5}$ and $10^{-8}$, respectively. In the last two columns of Table 3, there are two-tailed *p*-values calculated from the z-score tests for two population proportions, comparing the number of final solutions with an NLSE less than or equal to $10^{-5}$ and less than or equal to $10^{-8}$, respectively. Of the two *p*-values given for each formula, the upper one is for the test between the variants with numeric crossover and random mutation, while the lower *p*-value is for the test between the variants with numeric crossover and creep mutation. The bold values in the table indicate that the differences were not statistically significant at p<0.05.

The first observation that we can make from Table 3 is that there were only two cases where the use of numeric mutation produced more than half of the solutions with an NLSE lower than $10^{-5}$ (i.e., F17 and F20 using random mutation), and no such cases with an NLSE lower than $10^{-8}$. On the other hand, the proposed numeric crossover produced five and three such cases using NLSE limits of $10^{-5}$ and $10^{-8}$, respectively. Whereas this improvement did not seem to be very dramatic, the *p*-values were a different story. In exactly half of the comparisons (at NLSE of $10^{-5}$), numeric crossover was significantly better than one of the numeric mutations at p<0.05, and in almost one quarter of cases, it was better with p<0.01. The improvement was even better at a lower NLSE of $10^{-8}$ where more than half of the cases exhibited an improvement with statistical significance at p<0.01. Note that there were just two cases (i.e., F06 and F12) where numeric crossover was just slightly worse than creep mutation.

Table 4 shows the results obtained by C-RGEP. A quick comparison of Table 3 and Table 4 reveals to us that gene constraint indeed had a favorable effect on the behavior of the algorithm, as all three variants were better than their counterparts using the standard RGEP approach. Again, we were most interested in comparing the proposed numeric crossover with numeric mutations. This time, there was only a single case (i.e., F19) where numeric crossover performed slightly worse than creep mutation at an NLSE of $10^{-5}$. Otherwise, the overall improvement of numeric crossover at an NLSE of $10^{-5}$, with slightly more than half of improvements with statistical significance at p<0.05, was almost the same as it was with the standard RGEP algorithm. However, an improvement at a lover NLSE of $10^{-8}$ was quite better with C-RGEP. This time, 83.3% of cases were statistically significantly better using numeric crossover than they were using numeric mutation at p<0.05, and even at p<0.01, 73.8% were still better. Last but not least, the attained median test NLSE values were quite low with the proposed numeric crossover. In 23.8%, they were lower than $10^{-8}$, indicating a high potential that the proposed approach could create highly accurate constants.

### 5.1. Two Benchmark Cases

To round off our experimental work, we tested the three constant creation methods using two standard benchmark functions, Keijzer 14 and Korns 2, taken from the works by Keijzer [23] and Korns [22], respectively:

(10) f(x,y)=6sin(x)cos(y)[Keijzer14]

(11) f(x,y,z)=0.23+(14.2((x+y)/(3.0z))[Korns2]

**Figure 6:**
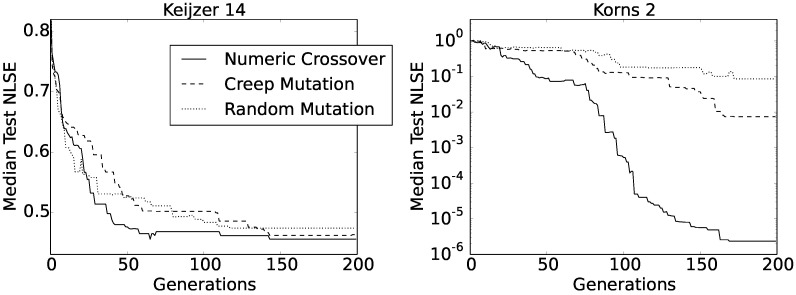
Convergence rate of the three constant creation methods on two standard benchmark functions using C-RGEP.

The first of the two functions only contains a single integer, two variables and has a depth of three grammar nodes. In this aspect, the function did not differ from our 21 test functions, and we ran the experiments with (10) under the same conditions as we did before. The Korns 2 test function, however, contains three variables and is four grammar nodes deep. For this reason, we extended the chromosome length to 31 genes and appended an extra variable to the terminal set. Furthermore, it turned out that a larger population was needed, so we increased the population size to 400. As in all previous experiments, we carried out 50 independent evolution runs for each of the two target formulas and every constant creation method, using the C-RGEP algorithm. Figure 6 shows the convergence curves of the obtained median test NLSEs as functions of generations.

At a first glance, there did not seem to be any difference between the three convergence curves obtained for the Keijzer 14 function, and also, the final values were very similar between all three methods (i.e., between 0.461 and 0.474). However, the shape of the curve obtained using numeric crossover had the most regular asymptotic shape. The same held for the Korns 2 function, only that this time, the curve was composed of two consecutive asymptotic shapes. Apart from this, numeric crossover produced much better solutions for the Korns 2 function, with the median test NLSE value well below $10^{-5}$.

We can find some additional information about these last experiments gathered in Table 5. Apart from the already familiar numbers of solutions having an NLSE better than $10^{-5}$ and $10^{-8}$, the table also reports minimum test NLSEs for each set of 50 runs. The results were particularly interesting for the Keijzer 14 function. Although the convergence rates of all three median test NLSEs were similar for this function, we can see from Table 5 that numeric crossover not only produced the highest number of accurate solutions, but also found some exact solutions (i.e., having an NLSE of zero). To be precise, among 50 runs, our method found the exact Keijzer 14 formula in four runs.

## 6. Discussions

In this paper, we presented a simple numeric crossover mechanism for use in RGEP. We have shown that the proposed technique is able to produce highly accurate solutions to certain symbolic regression problems, which the original RGEP method has many difficulties in solving even when using relatively efficient numeric mutations. We have seen that the improvement in the accuracy of solutions is more significant at a higher precision, and what is more, the improvement is even greater with C-RGEP, which itself exhibits a superior performance over the original RGEP. One of the reasons for the success of our method is the fact that the proposed numeric crossover operator aptly combines exploitation and exploration. One part of the numeric crossover (i.e., when the operator comes across two identical constants) is akin to a simple hill-climbing operator that automatically fine-tunes better solutions, which represents the exploration of the search space. Incidentally, we found out that there are a few percent of cases when two identical chromosomes participate as parents in crossover, in which case, the just mentioned mechanism is identical to creep mutation. The second part of the proposed numerical crossover (i.e., digit-wise crossover) works like an ordinary crossover and is therefore responsible for exploration of the search space.

We did not compare our method with any of the methods that use an external real parameter optimization method, even if these methods are known to produce most accurate results. That is because the methods that use a separate optimization tools are both complicated to implement and computationally expensive. That said, our aim was to develop a method that would be simple to implement and integrated into rather than added to the basic algorithm. Note that even much simpler and also often used numeric mutations, which we used for comparison, put certain additional computational strain on the algorithm, as they are often used as additional operations. Our method, on the other hand, does not impose any additional computational load on the algorithm, for the proposed crossover is integrated in the standard crossover operator of RGEP.

As a final remark, we would like to point out that we tested our constant creation method isolated on the most simple of all known GP variants (i.e., RGEP), not using any other approaches (not concerned with constant creation), such as separating individuals into so-called age-layers to prevent premature convergence or using subpopulations to preserve genetic diversity, which can significantly increase the search ability of an EA. Nevertheless, we believe we managed to show that our method is highly effective (at no additional computational cost) and that many researchers will readily try it with their solutions inasmuch as the method is extremely plain and should therefore present no difficulty in incorporating it into existing systems.

## Figures and Tables

**Table 1:** Genetic parameters used in our experiments.

Parameter	Description
Population Size	200
Generations	200
Function Set	{+, −, *, / (protected), sin, cos, tan, ln (protected),
	e*^x^*, x (protected), pass}
Terminal Set	{$0\leq C_{\mathrm{random}}\leq 10$, *x*} (for single-variable problems)
	{$0\leq C_{\mathrm{random}}\leq 10$, *x*, *y*} (for bivariable problems)
Constant Selection Probability	$p_{\mathrm{Tc}}=0.3p_{T}$ (see Equation (3)) ^1^
Operator Probabilities	$p_{\mathrm{singlePointCrossover}}=p_{\mathrm{twoPointCrossover}}=0.35$
	$p_{\mathrm{mutation}}=0.3$, $p_{\mathrm{rotation}}=0.5$^2^
Constant Creation	Random mutation, creep mutation or numeric crossover
Numeric Mutation Probability	0.2
Chromosome Length	15 genes ^3^
Selection	Tournament selection of size 3
Replacement Selection	Tournament selection of size 2
Elitism	Best individual always survives
Fitness	NLSE

^1^ Note that terminal selection probability $p_{T}$ always equals 1 when selecting a terminal in the case of gene constraint encoding. In that case, $p_{\mathrm{Tc}}=0.3$; ^2^ Rotation is always performed immediately after crossover, before the children are evaluated. A probability of 0.5 means that approximately half of the children produced by crossover are rotated; ^3^ In the case of gene constraint encoding, this number is obtained from a derivation tree depth of 3.

**Table 2:** List of problems used for testing different RGEP variants.

Label	Formula	Fitness/Test Cases
		(100 random points)
F01	$x^{2}-1.4981x+5.6517$	[−10,10]
F02	$1.7592x^{3}+1.7592$	[−10,10]
F03	$1.6190+x+9.8725/x^{2}$	[−10,10]
F04	1.7245x+8.1542xy	[−10,10]×[−10,10]
F05	9.8146−2.7770x/y	[−10,10]×[−10,10]
F06	1.6192+9.8815ln(x)	[0.001,20]
F07	−0.7391+3.7855sin(x)	[−10,10]
F08	0.1192+sin(0.6156x)	[−10,10]
F09	$-7.3914+0.1410e^{x}$	[−10,10]
F10	$-8.5410e^{2.1196x}$	[−10,10]
F11	9.5419+0.6720tan(x)	[−10,10]
F12	$-1.5193+9.1519\sqrt{x}$	[0,20]
F13	$-3.1871\sqrt{x}$	[0,20]
F14	6.5619x+2.1059cos(y)	[−10,10]×[−10,10]
F15	$0.1607/\sqrt{x}$	[0,20]
F16	2.9163ln(x)sin(x)	[0.001,20]
F17	4.4161xsin(y)	[−10,10]×[−10,10]
F18	$\sin(0.3165x^{2})$	[−10,10]
F19	0.5714x/ln(y)	[−10,10]×[0.001,20]
F20	3.1928+tan(x)−tan(y)	[−10,10]×[−10,10]
F21	$3.1817e^{\sin(x)}$	[−10,10]

**Table 3:** Results of the evolution of s-expressions using RGEP with two different numeric mutations and the proposed numeric crossover. Bold values indicate that the improvement is not statistically significant at p<0.05.

Formula	RGEP (Random Mutation)	RGEP (Creep Mutation)	RGEP (Numeric Crossover)	*p*-Value (NLSE $\leq 10^{-5}$)	*p*-Value (NLSE $\leq 10^{-8}$)
	Median Test NLSE				
	No. of Found Formulas (NLSE $\leq 10^{-5}$, NLSE $\leq 10^{-8}$)				
F01	0.00254	0.00258	0.00184	0.0349	0.0000
	(12,1)	(19,8)	(22,20)	0.5419	0.0076
F02	$1.90733\times 10^{-5}$	0.00140	$7.13315\times 10^{-6}$	0.0455	0.0001
	(19,1)	(17,5)	(29,16)	0.0160	0.0069
F03	0.00907	0.00095	$4.00492\times 10^{-5}$	0.0027	0.0000
	(9,1)	(15,8)	(23,17)	0.0989	0.0375
F04	$4.37925\times 10^{-5}$	$4.07221\times 10^{-5}$	$3.23353\times 10^{-5}$	0.1707	0.0000
	(10,0)	(7,3)	(16,14)	0.0324	0.0034
F05	0.01820	0.00974	0.00133	0.0005	0.0004
	(2,0)	(11,3)	(15,11)	0.3628	0.0209
F06	0.07677	0.02654	0.0052965	0.0251	0.0784
	(2,0)	(10,0)	(9,3)	0.8026	0.0784
F07	0.03154	0.01631	$3.13108\times 10^{-6}$	0.0000	0.0000
	(7,0)	(12,1)	(27,22)	0.0021	0.0000
F08	0.02996	0.03232	0.02669	0.0017	0.0114
	(0,0)	(6,0)	(9,6)	0.4009	0.0114
F09	0.00047	0.00028	0.00018	0.0375	0.0000
	(13,1)	(14,5)	(23,19)	0.0629	0.0010
F10	0.00012	0.00136	0.00036	0.5823	1.0000
	(7,0)	(4,0)	(9,0)	0.1362	1.0000
F11	0.13058	0.24147	0.027811	0.0001	0.0000
	(3,0)	(10,0)	(19,14)	0.0477	0.0000
F12	0.00595	0.02488	0.00620	0.0784	1.0000
	(0,0)	(4,1)	(3,0)	0.6965	0.3125
F13	0.05749	0.06368	0.03831	0.1835	0.0801
	(3,2)	(1,0)	(7,7)	0.0271	0.0061
F14	0.00073	0.00147	0.00052	0.1336	1.0000
	(7,0)	(10,0)	(13,0)	0.4777	1.0000
F15	0.12812	0.00109	$9.39478\times 10^{-6}$	0.0024	0.0000
	(14,1)	(22,13)	(29,25)	0.1615	0.0135
F16	0.01403	0.01637	0.01035	0.0836	0.0251
	(4,2)	(4,3)	(10,9)	0.0836	0.0643
F17	$2.59423\times 10^{-6}$	0.00010	$2.22545\times 10^{-12}$	0.0349	0.0000
	(28,6)	(24,21)	(38,38)	0.0040	0.0005
F18	0.77783	0.84318	0.58024	0.3077	0.1527
	(1,0)	(2,0)	(3,2)	0.6455	0.1527
F19	0.20179	0.21861	0.01328	0.0324	0.0009
	(7,3)	(5,3)	(16,16)	0.0069	0.0009
F20	$2.40286\times 10^{-6}$	0.02511	$1.27299\times 10^{-12}$	0.0375	0.0000
	(27,16)	(17,16)	(37,36)	0.0000	0.0000
F21	0.00606	0.02130	0.013325	0.1971	0.0001
	(13,3)	(17,13)	(19,19)	0.6745	0.1971

**Table 4:** Results of the evolution of s-expressions using C-RGEP with two different numeric mutations and the proposed numeric crossover. Bold values indicate that the improvement is not statistically significant at p<0.05.

Formula	C-RGEP (Random Mutation)	C-RGEP (Creep Mutation)	C-RGEP (Numeric Crossover)	*p*-Value (NLSE $\leq 10^{-5}$)	*p*-Value (NLSE $\leq 10^{-8}$)
	Median Test NLSE				
	No. of Found Formulas (NLSE $\leq 10^{-5}$, NLSE $\leq 10^{-8}$)				
F01	$2.92910\times 10^{-6}$	$2.06778\times 10^{-8}$	$8.81672\times 10^{-12}$	0.0007	0.0000
	(29,0)	(33,22)	(44,41)	0.0091	0.0001
F02	$8.20371\times 10^{-6}$	$7.64020\times 10^{-6}$	$7.32903\times 10^{-6}$	0.5353	0.0076
	(30,1)	(30,6)	(33,9)	0.5353	0.4009
F03	$4.45163\times 10^{-5}$	0.00649	$1.33771\times 10^{-5}$	0.3125	0.0000
	(20,0)	(14,6)	(25,21)	0.0238	0.0007
F04	$4.62953\times 10^{-5}$	$3.18061\times 10^{-5}$	$1.55923\times 10^{-10}$	0.0050	0.0000
	(16,0)	(23,10)	(30,28)	0.1615	0.0002
F05	0.00449	$2.32946\times 10^{-6}$	$1.03801\times 10^{-10}$	0.0000	0.0000
	(3,0)	(26,5)	(32,30)	0.2225	0.0000
F06	0.00543	0.03705	0.00610	0.0629	0.0032
	(5,0)	(3,0)	(12,8)	0.0117	0.0032
F07	0.03993	0.00023	$5.24175\times 10^{-9}$	0.0000	0.0000
	(8,0)	(19,2)	(29,26)	0.0455	0.0000
F08	0.02794	0.02883	0.02918	0.0220	0.0220
	(0,0)	(2,0)	(5,5)	0.2380	0.0220
F09	0.00021	0.00017	$1.83179\times 10^{-9}$	0.0093	0.0000
	(17,0)	(15,6)	(30,28)	0.0025	0.0000
F10	0.00037	0.00077	0.00014	0.0854	1.0000
	(7,0)	(9,0)	(14,0)	0.2340	1.0000
F11	0.23872	$1.71681\times 10^{-5}$	$1.83038\times 10^{-10}$	0.0000	0.0000
	(7,0)	(23,1)	(36,30)	0.0083	0.0000
F12	0.00913	0.01491	0.01200	0.1676	0.3125
	(1,0)	(2,0)	(4,1)	0.4009	0.3125
F13	0.00950	0.02490	$1.00544\times 10^{-5}$	0.0003	0.0000
	(8,2)	(15,12)	(25,21)	0.0414	0.0561
F14	0.00039	0.00046	$8.19655\times 10^{-5}$	0.0238	0.0011
	(14,1)	(14,2)	(25,12)	0.0238	0.0040
F15	$4.91555\times 10^{-7}$	$5.36922\times 10^{-7}$	$8.49969\times 10^{-12}$	0.5287	0.0000
	(31,7)	(26,22)	(34,34)	0.1031	0.0155
F16	0.00867	0.00921	0.00341	0.1471	0.0018
	(15,7)	(12,10)	(22,21)	0.0349	0.0173
F17	$3.97238\times 10^{-7}$	$2.09001\times 10^{-9}$	$2.45460\times 10^{-13}$	0.0324	0.0000
	(34,7)	(35,28)	(43,42)	0.0536	0.0022
F18	0.79627	0.98743	0.59888	0.0063	0.0009
	(3,0)	(7,1)	(13,10)	0.1336	0.0040
F19	0.01666	0.00045	0.00854	0.1416	0.0004
	(14,4)	(23,19)	(21,19)	0.6892	1.0000
F20	$5.21087\times 10^{-8}$	$7.30382\times 10^{-12}$	$2.39878\times 10^{-15}$	0.0001	0.0000
	(31,20)	(38,36)	(47,47)	0.0117	0.0034
F21	$4.72883\times 10^{-7}$	$4.59902\times 10^{-8}$	$1.90733\times 10^{-11}$	0.5093	0.0000
	(34,10)	(28,24)	(37,37)	0.0588	0.0076

**Table 5:** Results of the evolution of two benchmark s-expressions using C-RGEP with two different numeric mutations and the proposed numeric crossover.

Constant Creation Method	Keijzer 14				Korns 2		
	Minimum Test NLSE	NLSE $10^{-5}$	NLSE $10^{-8}$		Minimum Test NLSE	NLSE $10^{-5}$	NLSE $10^{-8}$
Random mutation	$3.642\times 10^{-10}$	10	2		$1.242\times 10^{-9}$	11	1
Creep mutation	$3.855\times 10^{-13}$	12	10		$5.761\times 10^{-10}$	20	6
Numeric crossover	0.000	16	14		$2.337\times 10^{-13}$	30	20

## Abbreviations

The following abbreviations are used in this manuscript:

C-RGEP	Constrained RGEP (i.e., RGEP with gene constraint)
EA	Evolutionary Algorithm
ERC	Ephemeral Random Constant
GA	Genetic Algorithm
GE	Grammatical Evolution
GEP	Gene Expression Programming
GP	Genetic Programming
NLSE	Normalized Least Squares Error
RGEP	Robust Gene Expression Programming
RPN	Reverse Polish Notation
SR	Symbolic Regression
